# Exploring the Proteomic Landscape and Immunomodulatory Functions of *Edwardsiella piscicida* Derived Extracellular Vesicles

**DOI:** 10.4014/jmb.2410.10001

**Published:** 2024-12-17

**Authors:** Mawalle Kankanamge Hasitha Madhawa Dias, E.H.T. Thulshan Jayathilaka, Jayasinghage Nirmani Chathurangika Jayasinghe, Nipuna Tennakoon, Chamilani Nikapitiya, Ilson Whang, Mahanama De Zoysa

**Affiliations:** 1 College of Veterinary Medicine and Research Institute of Veterinary Medicine, Chungnam National University, Daejeon 34134, Republic of Korea; 2 National Marine Biodiversity Institute of Korea (MABIK), Janghang-eup, Seocheon 33662, Republic of Korea

**Keywords:** *Edwardsiella piscicida*, extracellular vesicles, immunomodulation, proteomic analysis, Raw 264.7 cells, Zebrafish (*Danio rerio*)

## Abstract

Extracellular vesicles (EVs) have garnered attention in research for their potential as biochemical transporters and immune modulators, crucial for regulating the host immune system. The present study was conducted to isolate and characterize EVs from Gram negative bacteria *Edwardsiella piscicida* (*Ep*EVs) and investigate their proteomic profile and immune responses. Isolation of *Ep*EVs was carried out using ultracentrifugation method. Transmission electron microscopy results confirmed the spherical shape of *Ep*EVs. The average size and zeta potential were 85.3 ± 1.8 nm and -8.28 ± 0.41 mV, respectively. *Ep*EVs consisted of 1,487 distinct proteins. Subcellular localization analysis revealed that “cell” and “cell part” were the most predominant areas for protein localization. Proteins associated with virulence, along with several chaperones that facilitate protein folding and stability, were also present. No toxicity was detected when *Ep*EVs were treated to fathead minnow (FHM) cells up to 100 μg/ml. Fluorescent-labeled *Ep*EVs showed cellular internalization in FHM cells at 24 h post treatment (hpt). *In-vitro* gene expression in Raw 264.7 cells showed upregulation of interleukin *(Il)6*, *Il1β*, and interferon *(Ifn)β* with simultaneous upregulation of anti-inflammatory *Il10*. *In vivo*, gene expression revealed that except for heat shock protein *(hsp)70*, all other genes were upregulated suggesting that *Ep*EVs induced the expression of immune-related genes. Western blot analysis showed increased protein levels of tumor necrosis factor (Tnf)α in *Ep*EVs-treated spleen tissue of zebrafish. Our results confirm that *Ep*EVs can be successfully isolated using the ultracentrifugation method. Furthermore, exploring immunomodulatory mechanism of *Ep*EVs is essential for their potential use as novel therapeutics in fish medicine.

## Introduction

Extracellular vesicles (EVs) have emerged as a promising biomaterial in modern therapeutics, particularly in the development of immunotherapy and targeted drug-delivery systems [1]. Like eukaryotes, bacteria also produce EVs, known as bacterial EVs (BEVs), which remain a relatively underexplored area with vast untapped potential. BEVs can deliver both natural and bioengineered cargo [2]. These nanovesicles, typically ranging from 20-400 nm in diameter, are produced from both gram positive and gram negative bacteria [2]. These spherical-shaped vesicular bodies contain a diverse composition of molecules, including structural components like lipopolysaccharide (LPS) and peptidoglycan, toxins, periplasmic and cytoplasmic proteins, and nucleic acids [2-4]. Biogenesis of gram negative BEVs is mainly explained by two proposed models; outer membrane blebbing and explosive cell lysis [2]. In the outer membrane blebbing model, instability in the peptidoglycan layer causes the outer membrane to bud into the periplasm, while explosive cell lysis occurs when the inner membrane protrudes into the periplasm through weak points in the peptidoglycan layer [2]. Following the release, BEVs have the potential to be internalized by both prokaryotic and eukaryotic cells, where they can deliver their cargo to modulate complex biological processes and facilitate intercellular communication [5].

Both pathogenic and non-pathogenic bacteria can produce BEVs, with varying functions depending on their cargo, type of bacteria, and its target. These vesicle components play roles in various biological processes, including the expression of virulence, phage infection, cellular metabolites transportaiton, and horizontal gene transfer [2]. Furthermore, it has been shown that BEVs exhibit protein cargo sorting and selective loading [6]. This cargo selectivity is closely linked with the biogenesis process, indicating that it is a highly regulated and orchestrated biological phenomenon rather than a random occurrence [2]. For the selective cargo loading, electrostatic interactions, curvature-recognizing protein interactions, and lipoprotein interactions work tandemly during BEVs biogenesis [4]. Furthermore, research by Chen *et al*. in *Shewanella vesiculosa* proposed the “P_49_ protein secretion mechanism”, suggesting that foreign protein can be incorporated into BEVs via fusing with the P_49_ protein [7]. Therefore, understanding BEVs proteins and their interactions with host cells is crucial for discovering disease biomarkers, which can be applied in diagnostics, screening, prognosis, and treatment [8]. In-depth knowledge of these cargo molecules in specific bacteria could broaden our knowledge of their functional roles in relation to each host. Proteomics is a valuable approach that aids researchers in identifying and elucidating BEVs-host interactions, making it a powerful tool for disease diagnostics [9]. Although several studies exist on mammalian pathogenic BEVs, research on fish pathogenic BEVs remains a relatively unexplored subject area. Table 1 summarizes the available proteomic studies on fish pathogenic BEVs and their activities.

*Edwardsiella piscicida* is a gram negative, rod-shaped bacterium that is a well-established pathogen in both freshwater and marine fish [15-17]. Additionally, *E. piscicida* poses a serious risk to human health as the consumption of contaminated food products can lead to cross-species transmission of the infection to humans [15]. Moreover, the pathogenesis of *E. piscicida* is largely driven by its Type III and Type VI secretion systems (T3SS and T6SS), which are crucial virulence factors and key targets for vaccine development against edwardsiellosis [15]. Although a single study has explored the potential of *E. tarda* EVs as a vaccine candidate, the present study provides a broader approach by focusing on isolation of BEVs from *E. piscicida* using the ultracentrifuge method, along with the characterization of bacterial marker proteins. Moreover, this study includes a comprehensive proteomic analysis of *Ep*EVs, as well as assessments of their toxicity and immunomodulatory effects in Raw 264.7 cells and zebrafish.

## Materials and Methods

### *E. piscicida* Culture and Isolation of EVs

A loopful of *E. piscicida* (lab strain) was streaked onto Brain Heart Infusion (BHI) plates supplemented with 1%NaCl and incubated overnight at 25°C. A single colony was picked and inoculated into BHI+1% NaCl broth (4 ml), followed by overnight incubation at 25°C with continuous agitation at 180 rpm.

For EVs isolation, a large culture (1 L) of *E. piscicida* was prepared by adding an overnight-grown bacteria culture (OD_595_ ~ 1.0) into BHI+1% NaCl broth at 0.5% v/v and incubated for 24 h. Following the bacteria centrifugation, the supernatant was filtered (0.2 μm) and concentrated (10×) using 100 kDa Amicon^®^ Ultra-15 centrifugal filters (Millipore, Cork, Ireland). The supernatant was ultracentrifuged using a CP100NX UC system (Koki Holdings Co. Ltd., Japan) equipped with a P28S2 swinging rotor (Himac, Ibaraki, Japan) at 100,000 ×*g*; 4°C for 3 h. The resulting *Ep*EVs pellet was washed using ultracentrifuged and filtered phosphate-buffered saline (PBS) (Gibco-BRL, USA) following the same aforementioned conditions. Pelleted *Ep*EVs were dissolved in filtered PBS (900 μL) and kept at -80°C until further study.

### Characterization of *Ep*EVs

**Biogenesis, Morphology, Particle Size, Concentrations, and Surface Charge**

*Ep*EVs biogenesis was examined with field emission scanning electron microscopy (FE-SEM) following a pre-described method [18]. Briefly, bacterial cell pellet from an overnight grown and reinoculated fresh *E. piscicida* culture was washed with PBS and dehydrated using a gradient ethanol series (30, 50, 70, 80, 90, and 100%) and coated with platinum. Finally, observation was done using FE-SEM (Serion FEI, Netherlands).

The Morphology of *Ep*EVs was evaluated using FE-transmission electron microscopy (FE-TEM) opting for a Tecnai G2 F30 super-twin; FEI system (Hillsboro, USA) according to a method described previously [19]. Particle size and distribution were assessed by nanoparticle tracking analysis (NTA) using a NanoSight NS300 system (Malvern Technologies, UK) while the surface charge of *Ep*EVs was measured by zeta potential analyzer (Zetasizer Nano ZSP, UK) according to the method described by Nikapitiya *et al*. [19].

### Protein Profile and Molecular Markers of *Ep*EVs

The protein content of isolated *Ep*EVs was quantified using the Bradford assay (Bio-Rad, USA) according to the manufacturer’s instructions. For both *in vitro* and *in vivo* treatments, *Ep*EVs were quantified based on the protein content obtained from this assay. The protein profile of *Ep*EVs was determined using 12% sodium dodecyl sulfate-polyacrylamide gel electrophoresis (SDS-PAGE) as described previously [20]. *Ep*EVs sample (25 μg) were mixed with 4 × Laemmli sample buffer (Bio Rad) and denatured at 95°C for 10 min. The sample was loaded onto Mini-PROTEAN TGX gels (Bio Rad) and electrophoresed at 100 mV for 90 min. Protein bands were visualized by Coomassie blue staining (Biosesang, Republic of Korea).

For Western blot analysis, *Ep*EVs proteins were separated on an SDS-PAGE gel and transferred to Immobilon-P Polyvinylidene difluoride membranes (Millipore Corporation, USA). Membranes were blocked with 5% (w/v) bovine serum albumin (BSA) prepared in Tris-buffered saline with 0.05% Tween 20 (TBST) (LPS Solution, Republic of Korea) at 26 ± 2°C for 1 h. They were then incubated overnight at 4°C with primary antibodies against flagellin (Abcam, Republic of Korea) and *E. coli* outer membrane protein A (OmpA) (Antibody Research Corporation, USA). After washing with PBS containing 0.05% Tween 20 (PBST) (three times), membranes were incubated with secondary antibodies (Table 2) at 26 ± 2°C for 1 h. Protein bands were visualized using the Western Femto ECL Kit (LPS solution, Republic of Korea) and detected with a chemiluminescence imaging system (Fusion Solo S, Vilber, France).

### Protein Preparation and HPLC-MS/MS Based Proteomic Analysis

Proteomic analysis of *Ep*EVs was conducted as described in our previous publication [21]. Briefly, *Ep*EVs samples were treated with a reducing agent dithiothreitol (DTT; 10 mM) at 37°C for 30 min followed by incubation with iodoacetamide (IAM; 55 mM) at 25°C for 45 min in dark. After processing, samples were centrifuged (25,000 ×*g* at 4°C for 15 min) and quantified using the Bradford assay (Bio-Rad), and subjected to electrophoresis for protein separation.

During the enzymatic digestion process, 100 μg of protein solution was mixed with 50 mM ammonium bicarbonate (NH_4_HCO_3_) to make final SDS concentration below 0.1%. Trypsin (2.5 μg) was added at a protein-to-enzyme ratio of 20:1 and incubated at 37°C for 4 h for digestion. The resulting peptides were desalted using a Strata X column (10 mg/ml, Tubes; Phenomenex, USA) and dried under vacuum using a MaxiVac beta freeze dryer (LaboGene™, Bjarkesvej, Denmark). Peptide separation was carried out using a Shimadzu (LC-20AB) high-performance liquid chromatography (HPLC) system equipped with a Gemini C18 column (4.6 mm internal diameter, 5 μm column size, 25 cm column length). Separation was performed at a flow rate of 1 ml/min and 10 fractions were collected and subsequently lyophilized.

*Ep*EVs were analyzed for peptide sequencing and quantification using Orbitrap Exploris 480 mass spectrometer system (Thermo Fisher Scientific, USA) coupled to an UltiMate 3000 UHPLC system (Dionex, USA). The separated peptides were ionized using a nano-electrospray ionization (ESI) source and analyzed in data-dependent acquisition (DDA) mode. Peptide samples were reconstituted, centrifuged, and separated using a defined gradient at a flow rate of 500 nL/min. MS parameters were optimized to ensure effective peptide sequencing and protein quantification, including ion source voltage, scanning range, resolution, collision energy, and precursor peptide ion selection criteria.

### Bioinformatics of Proteomic Data

In the DDA library construction, Mascot software (version 2.3.02) was employed to identify peptides from protein sequences in an *Edwardsiella*-specific database. Trypsin was employed for protein cleavage with allowance for up to one missed cut. The peptide-protein matching error threshold was set at 1% and the minimum acceptable peptide length was seven amino acids. Fixed modifications included carbamidomethyl at cysteine sites, while variable modifications included methionine oxidation, conversion of glutamine to pyroglutamate, and deamidation of pyroglutamate.

To identify protein groups, tandem MS results were pre-processed and re-scored using Percolator software to improve the matching accuracy. The data were filtered with a false discovery rate (FDR) of 1% at the spectral level, yielding a list of significant spectra and peptides. Gene ontology (GO) annotation was performed using the DAVID database (https://david.ncifcrf.gov/) accessed on 2023.12.20 under default settings to assess cluster enrichment. Pathway enrichment analysis was conducted using the Kyoto Encyclopedia of Genes and Genomes (KEGG), while protein-protein interaction (PPI) predictions of *Ep*EVs and *E. tarda* were conducted using the Search Tool for the Retrieval of Interacting Genes/Proteins (STRING) database (Version 12.0; https://string-db.org) at default settings (accessed on 2024.04.26). These analyses provided valuable insights into the functional roles and interaction networks of the profiled proteins in the BEVs.

### Assessment of *In Vitro* and *In Vivo* Toxicity of *Ep*EVs

Fathead minnow (FHM) epithelial cells and Raw 264.7 murine macrophage cells were used to determine the toxicity and safety dose of *Ep*EVs. FHM cells were cultured in sealed cap culture flasks at 25°C, using L15 medium supplemented with 10% fetal bovine serum (FBS) (Wellgene Co., Ltd., Republic of Korea). Raw 264.7 cells were maintained in a Dulbecco's Modified Eagle Medium (DMEM) (Wellgene Co., Ltd., Republic of Korea) supplemented with 10% FBS and 1% antibiotic mixture (Penicillin/Streptomycin) (Gibco, USA).

The cytotoxicity of *Ep*EVs was assessed using the Cellrix Viability Assay Kit (MediFab, Republic of Korea) following the manufacturer's instructions. FHM and Raw 264.7 cells were seeded in a 96-well plate at 2 × 10^5^ cells/ml (100 μl/well) and incubated at 25°C and 37°C for 12 h, respectively. *Ep*EVs (10 μl; concentrations ranging from 0 to 100 μg/ml) were treated to the cells and incubated for 24 h, after which Cellrix reagent (10 μl per well) was introduced. After incubation at 37°C for 2.5 h, the optical density at 450 nm (OD_450_) was measured using a microplate reader (Bio Rad, Japan). Based on the results, the safety dose was determined. Reactive oxygen species (ROS) generation was measured in seeded Raw 264.7 cells following a 24 h incubation. The cells were treated with 2'7'dichlorodihydro-fluorescein diacetate (DCFHDA) (Sigma-Aldrich, USA) at a concentration of 5 μg/ml for 30 min. Excess dye was removed by washing with PBS and ROS generation was observed under a fluorescence microscope (DMi8; Leica, Germany).

Adult zebrafish were reared, bred, and embryos were collected following established procedures [22]. Healthy larvae [60 h post-fertilization (hpf)] were selected for the experiment and treated with *Ep*EVs (10-100 μg/ml; 10 larvae per treatment) for 96 h. Mortality was monitored every 12 h. After 96 h of treatment, five larvae from each group were incubated with DCFHDA (5 μg/ml) for 30 min to assess *Ep*EVs-induced ROS generation, following a previously published method [22]. Excess dye was removed by washing with PBS, and imaging was performed using a Leica S8 APO stereo microscope (Nikon SMZ100, Japan) equipped with a SFA fluorescent filter (NIGHTSEA, USA).

### Fluorescent Labeling and Internalization of *Ep*EVs in FHM Cells

Fluorescent labeling of *Ep*EVs was conducted to confirm cellular uptake using fish cells. The procedure followed the guidelines provided by the ExoSparkler kit (Dojindo Molecular Technologies Inc., USA) for the fluorescent labeling of *Ep*EVs. Briefly, *Ep*EVs (10 μg) were diluted in PBS and treated with Mem dye-green solution (2 μl). The mixture was incubated for 30 min, then transferred to a filtration tube and centrifuged at 3,000 ×*g* at 26 ± 2°C for 5 min. The samples were washed twice with 100 μl of PBS each time, followed by re-centrifugation under the same conditions. Finally, the labeled *Ep*EVs were reconstituted in PBS.

Pre-seeded FHM cells (1.25 × 10^4^ cells/ml) in 12 well plates (1 ml/well) were treated with fluorescent-labeled *Ep*EVs (20 μg in 50 μl) for 24 h. Subsequently, the cells were washed with PBS and stained with 4',6-diamidino-2-phenylindole (DAPI; 300 nM for 5 min) (Sigma-Aldrich). *Ep*EVs internalization was then observed using a fluorescence microscope.

### Analysis of *In Vitro* Immunomodulatory Activity of *Ep*EVs in Raw 264.7 Cells

Raw 264.7 cells (1 × 10^5^ cells/well) were seeded in 6-well plates and incubated at 37°C. After 12 h, the cells were treated with *Ep*EVs at two different concentrations (5 and 10 μg/ml) for 24 h, after which they were harvested. Total RNA was isolated using the NucleoSpin RNA Mini Kit (Macherey-Nagel, Germany). RNA concentrations were measured using a NanoDrop One spectrometer (Thermo Fisher Scientific), and cDNA was synthesized using the PrimeScript 1^st^ strand cDNA Synthesis Kit (TaKaRa, Japan). Gene-specific primers for quantitative real-time PCR (qRT-PCR) are in Table 3. The cDNA was diluted to 10 ng/ml and qRT-PCR was performed using the Thermal Cycler Dice Real-Time System (TaKaRa). The relative mRNA expression of each gene was analyzed using the 2^−ΔΔCT^ method with *Gapdh* serving as the housekeeping gene [23].

### Analysis of Immune Gene and Protein Expression in *Ep*EVs-Treated Zebrafish

Adult zebrafish (average body weight of 0.45±0.05g) were acclimatized for two weeks before being intraperitoneally injected with *Ep*EVs (5 and 10 μg/fish), following a protocol in a previous publication [23]. Kidney and spleen tissues (3 kidneys per replicate) from each group were collected at 6 and 24 h post-treatment (hpt) and stored at -80°C. For immune gene expression analysis, RNA was extracted from kidney tissues and cDNA was synthesized according to the manufacturer's protocols. Zebrafish gene-specific primers are listed in Table 3, with *β-actin* used as the housekeeping gene. For protein expression analysis, spleen tissue was used. Tissues were lysed using ProEX CeTi Lysis buffer (TransLab, Republic of Korea) and the total protein content was quantified using the Bradford method. After preparing the samples, 25 μg of protein from each sample was loaded into a 12% SDS-PAGE gel, and immunoblotting was performed. Details of used primary antibodies and corresponding secondary antibodies are provided in Table 2. Finally, the membranes were incubated and visualized using a chemiluminescence detection system.

### Statistical Analysis

The significance between each group was determined using one-way analysis of variance (ANOVA) with GraphPad Prism software version 8 (USA). Mean separation was conducted using an unpaired two-tailed *t*-test, and statistical significance was defined as *p* < 0.05.

## Results

### Isolation and Characterization of *Ep*EVs

**Fig. 1:**
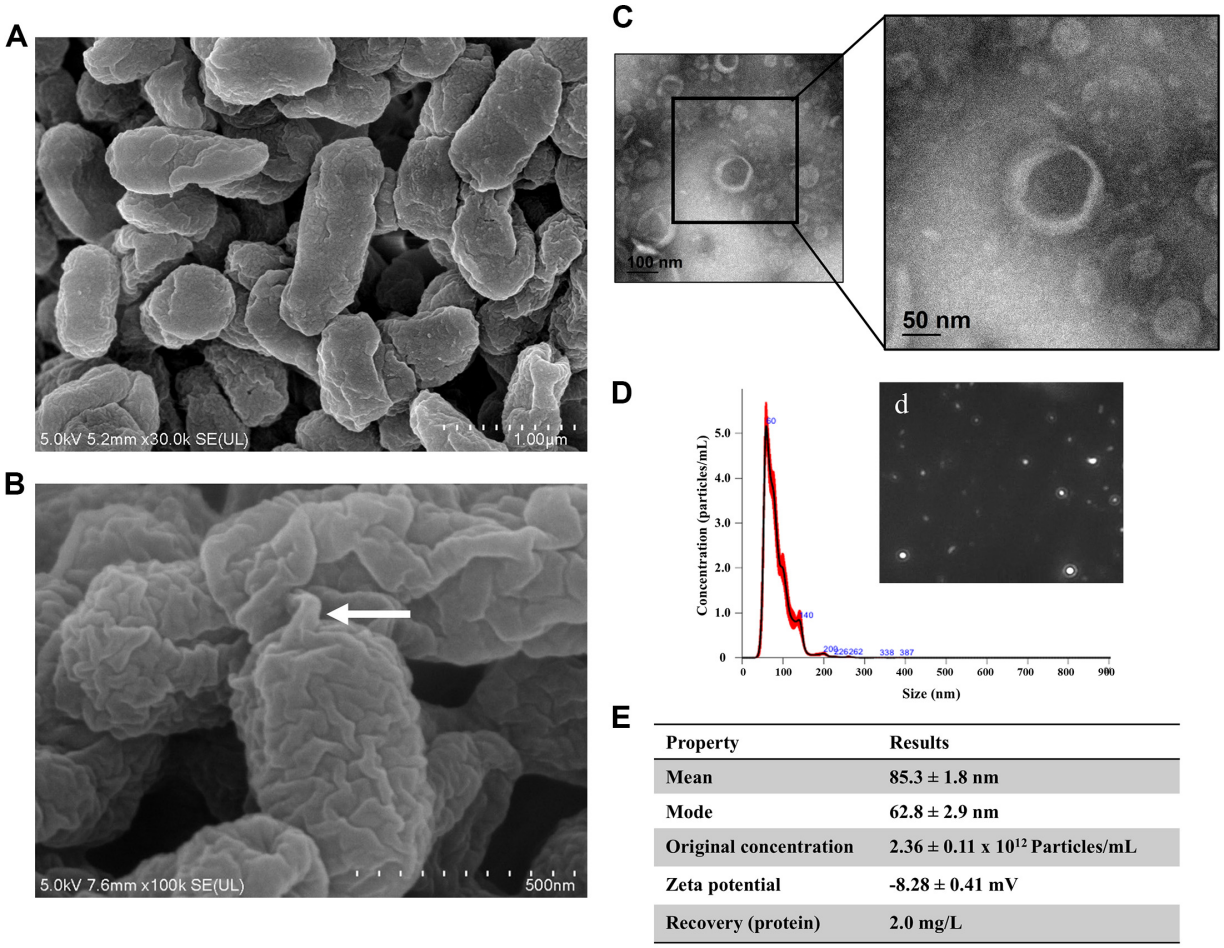
*Ep*EVs characterization. (**A**) Visualization of morphological features and (**B**) EVs release process from *E. piscicida* captured using FE-SEM. The white arrow indicates the formation of a bulbous structure preceding EVs release. (**C**) FE-TEM analysis of *Ep*EVs displaying a spheroid double membrane-bound morphology at different magnifications (100 and 50 nm). (**D**) Nanoparticle tracking analysis quantified the particle size and concentration of *Ep*EVs, with a graph depicting concentration plotted against particle size. A still frame from the particle distribution video is shown (d). (**E**) Comprehensive characterization of *Ep*EVs, including particle size distribution (mean and mode), original concentration, zeta potential, and total protein recovery.

Initially, we conducted molecular identification and FE-SEM analysis to confirm that the selected bacterium was *E. piscicida*. 16S rRNA gene sequencing results were analyzed using the EzBioCloud 16S database (CJ Bioscience, Inc., Republic of Korea) and showed 100% similarity (as of 2023.03.10) to the 16S rRNA genes of known *E. piscicida*. This, in turn, aids in isolating EVs specifically from *E. piscicida*. FE-SEM imaging displayed a prominent rod-shaped bacterium resembling the morphology of *E. piscicida* (Fig. 1A). When examining *E. piscicida* under higher magnification (×100K), spherical structures were observed that attached to the outer membrane of the bacterial surface, providing insights into the biogenesis of *Ep*EVs (Fig. 1B). Using FE-TEM imaging, *Ep*EVs were identified as small, spherical bilayer membrane structures (Fig. 1C). NTA-based size distribution analysis of *Ep*EVs revealed that a larger proportion of particles falls within the 50-150 nm size range (Fig. 1D). Moreover, the visual representation of particles showed evenly distributed *Ep*EVs, in the sample (Fig. 1d). Percentile values showed that 90% of the particles were smaller than 128.5 ± 3.7 nm, while only 10% were smaller than 55.5 ± 0.9 nm (data not shown). Furthermore, the mean particle size was 85.3 ± 1.8 nm (Fig. 1E). The concentrations of *Ep*EVs were 2.36 ± 0.11 × 10^12^ particles/ml, while the protein recovery was 2 mg/l of bacterial culture. Zeta potential analysis further revealed a negative surface charge of *Ep*EVs (-8.28 ± 0.41 mV).

### Total Protein Profile and Molecular Characterization of *Ep*EVs

**Fig. 2:**
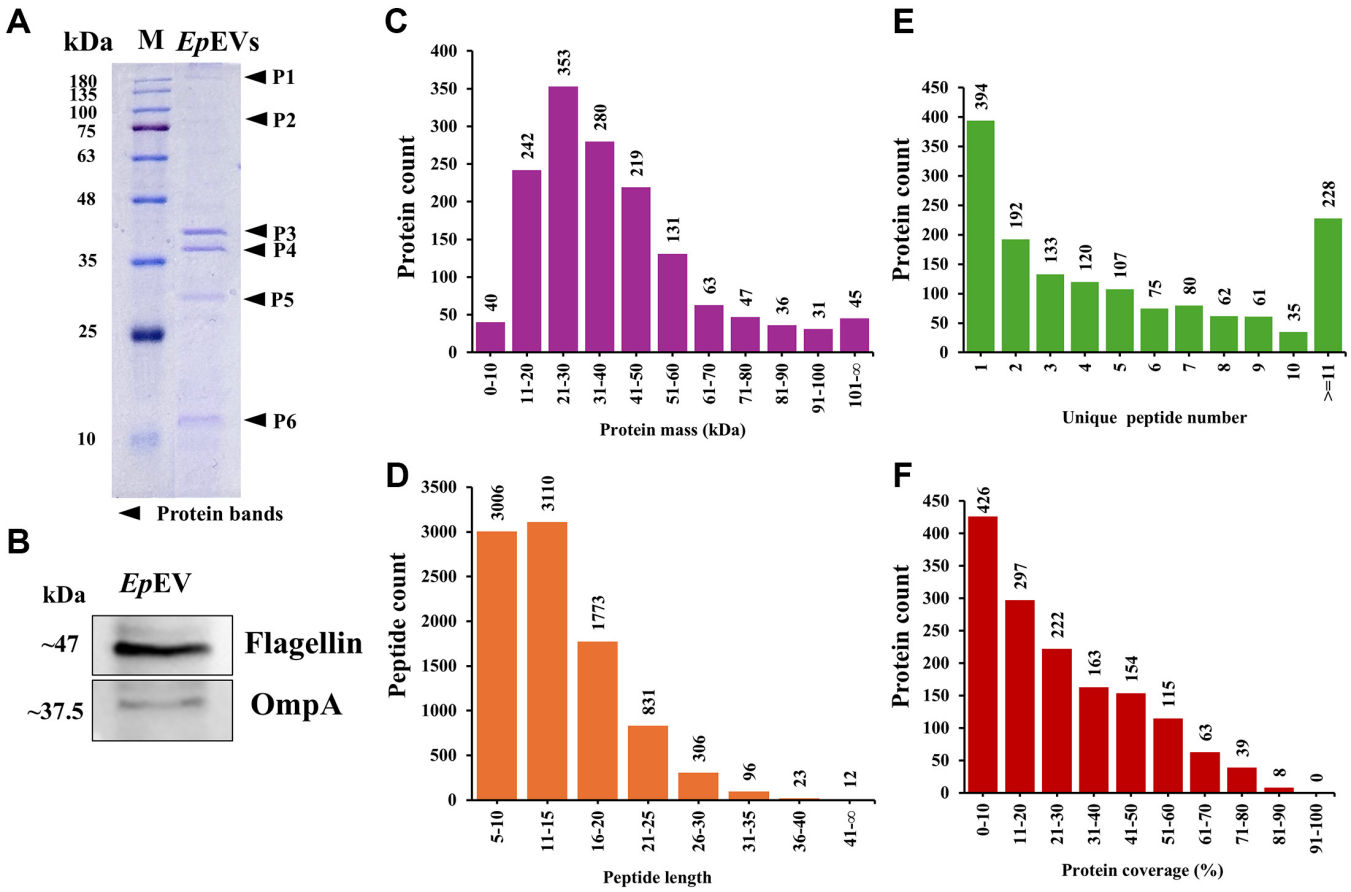
Protein profile and characteristics of *Ep*EVs. (**A**) SDS-PAGE gel stained with Coomassie blue shows distinct protein bands (P1-P6) indicated by black arrows. (**B**) Western blot analysis identified BEVs marker proteins. *Ep*EVs (25 μg total protein) were loaded into an SDS-PAGE gel (12%) and transferred to a membrane. Membranes were incubated overnight with polyclonal primary antibodies (Flagellin and OmpA) followed by relevant secondary antibodies. BioFACT triple-color protein marker (10-180 kDa) was used as a reference. Proteomic analysis further characterized the total proteome of *Ep*EVs. (**C**) Protein mass distribution, (**D**) peptide length distribution, (**E**) unique peptide distribution, and (**F**) protein coverage distribution. Peptides extracted from *Ep*EVs were fractionated into 10 fractions, further separated using HPLC, and analyzed using MS/MS for protein identification.

Understanding the total protein profile and molecular characteristics of BEVs is essential for elucidating their biological functions. The total protein profile of *Ep*EVs was examined using Coomassie blue-stained SDS-PAGE, highlighting the major protein components and their relative abundances (Fig. 2A). Six prominent protein bands were observed in the range of 10-180 kDa, with the highest band intensity localized between the molecular mass range of 35-48 kDa. Western blotting was conducted to detect the presence of BEVs marker proteins (Fig. 2B). Flagellin and OmpA were expressed at approximately 47 and 37.5 kDa, respectively and the intensity of the flagellin band was greater than that of OmpA. This finding was further supported by *Ep*EVs proteomic analysis which showed higher intensity-based absolute quantification (iBAQ) values for flagellin isomers compared to OmpA isoforms (Table S1).

### Proteomic Analysis of *Ep*EVs

We conducted a proteomic analysis of *Ep*EVs to study the entire proteome using HPLC-MS/MS. Raw mass spectrometry data were searched against the Mascot MS/MS database for peptide identifications and resulted in a total spectra of 56,179 (Table S2). Next, a subset of the total spectra was denoted as identified spectra of 15,911, which were matched with known spectra. This resulted in 10,745 identified peptides including unique peptides of 9,157 (85.22%). After searching the *Edwardsiella*_qc database (6,957 total sequences), we identified 1,487 proteins from *Ep*EVs. The most abundant protein mass range was 20-30 kDa (353 proteins) whereas the least number of proteins (31) had a molecular mass range of 90-100 kDa (Fig. 2C). In peptide length distribution analysis, the highest spike was noted for the 11-15 peptide length range (3110 peptides) (Fig. 2D). In the peptide length range of 20 – infinity (∞), only 13.85% of peptides are represented, where the highest was observed between 21-25 (831). Unique peptide distribution revealed about 51.64% of the identified proteins had three or more peptides while about 73.50% of the identified proteins had 2 or more peptides (Fig. 2E). About 71.35% and 51.38% of peptides showed more than 10 and 20% sequence coverage, respectively (Fig. 2F) which suggested that a higher amount of peptides had a lower percentile coverage.

**Fig. 3:**
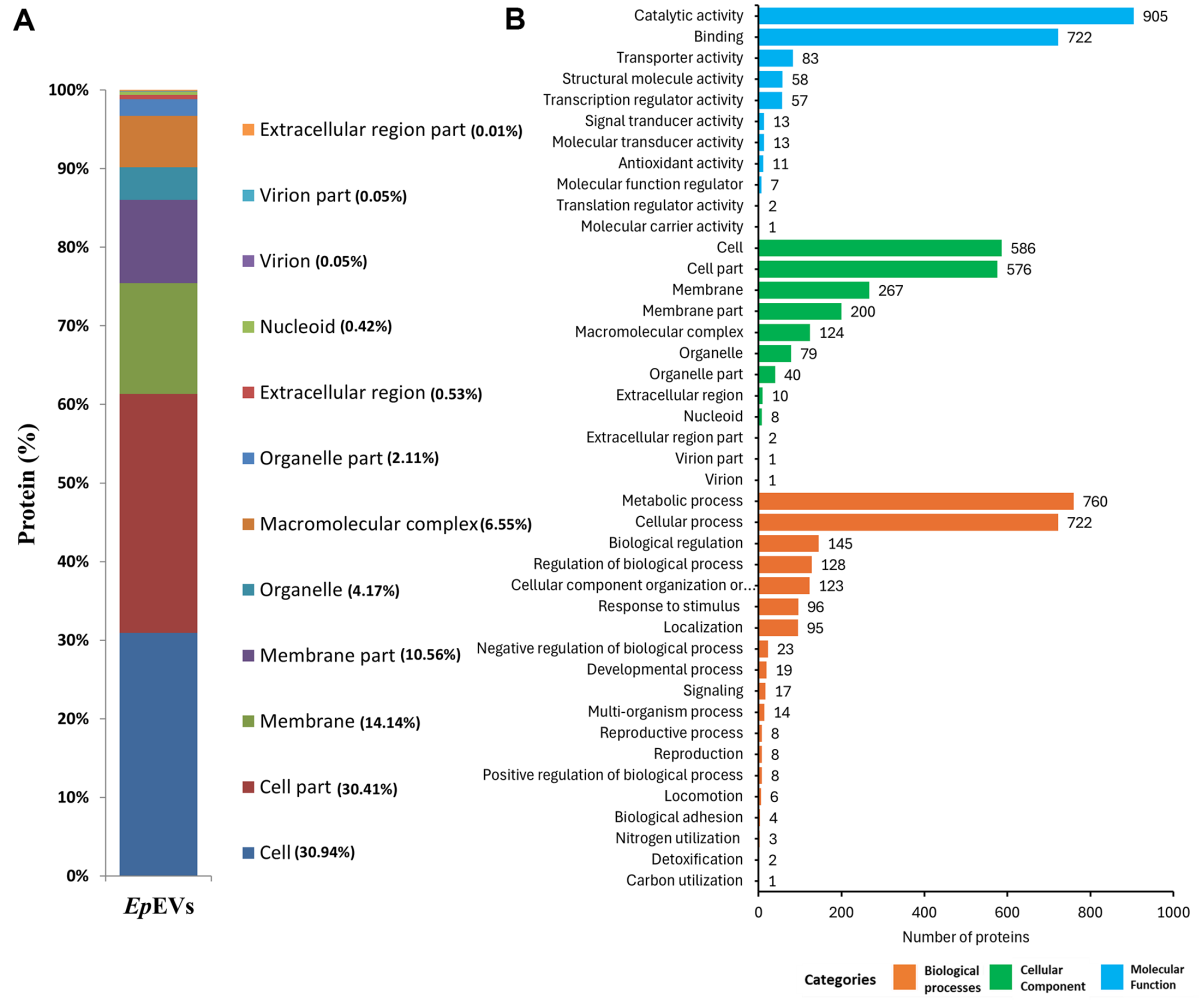
Localization and functional annotations of *Ep*EVs. (**A**) Subcellular localization of *Ep*EVs proteins was determined following HPLC-MS/MS analysis to identify the proteome in *Ep*EVs. (**B**) Gene ontology (**GO**) analysis was conducted to explore the various functional roles of the proteins identified through proteomic analysis.

To find out the physiological contexts, interactions, and regulatory roles of *Ep*EVs, subcellular localization of identified proteins was predicted. As expected the majority of protein localization was classified under ‘cellular component’ (30.94%). Furthermore, almost similar protein numbers (30.41%) represented the ‘cell part’ which together comprised 61.35% of the total proteins (Fig. 3A). ‘Extracellular region’ (0.01%) was the least number of proteins present in *Ep*EVs. Furthermore, GO analysis was conducted to map the molecular functions for the identified proteins categorizing them into three groups namely, biological processes, cellular components, and molecular functions (Fig. 3B). The majority of the proteins belonged to molecular functions where ‘catalytic activity’ related proteins such as Chorismate mutase, Cytochrome c-type protein, Glutathione synthetase, etc. were highly abundant (905). Proteins related to ‘binding’ (HTH-type transcriptional regulator IscR, Ribosomal RNA small subunit methyltransferase B, etc.) were the second highest out of the category (722). ‘Cell and cell part ’-related proteins belonging to the cellular component category were 586 and 576, respectively. Biological processes regulating proteins are annotated into 19 functional groups and out of them ‘metabolic processes’ [Ribonuclease G, 23S rRNA (guanosine-2'-O-)-methyltransferase RlmB, etc.] and ‘cellular processes’ (Signal peptide peptidase SppA, Macrodomain Ter protein, etc.) regulating proteins were the top two categories (760 and 722, respectively) while only one protein (Aldehyde-alcohol dehydrogenase) was recorded for ‘carbon utilization’ which was shown to be the least. Furthermore, from the identified proteins various virulence factors, such as proteins related to Type III secretion system (EscV/YscV/HrcV family type III secretion system export apparatus protein, TyeA family type III secretion system gatekeeper subunit, and CesD/SycD/LcrH family type III secretion system chaperone), flagellin and Omps and their assemblies, multiple chaperone proteins (HtrA protease/chaperone protein, Chaperone protein DnaJ, Heat shock chaperone IbpB), etc. were also present (Table 4). From the observed results it is apparent that the identified *Ep*EVs proteins have a vast array of functional properties that could be a considerable advantage for using it as an immunomodulating agent or as a vaccine candidate.

**Fig. 4:**
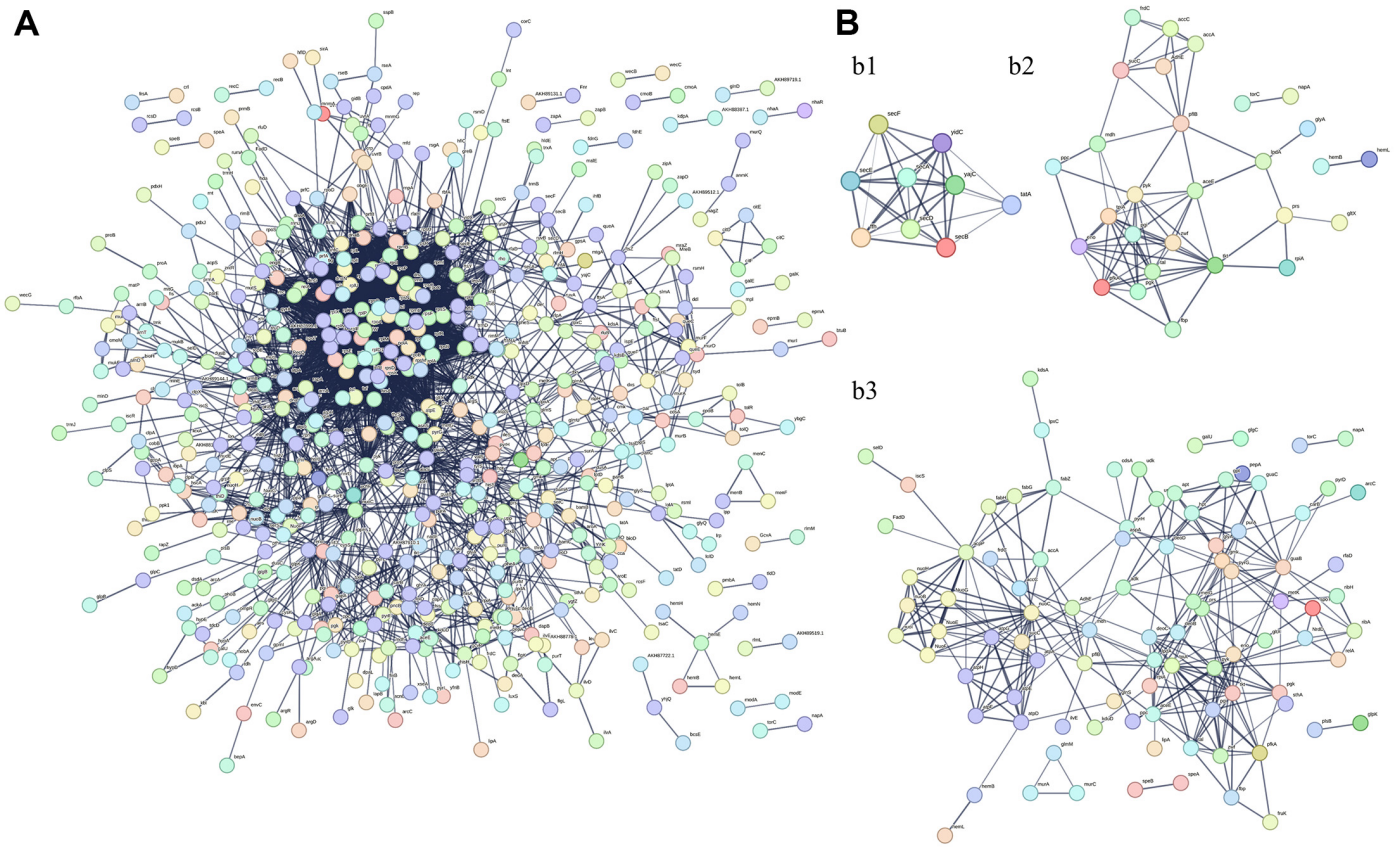
Protein-protein interaction (**PPI**) network prediction between *Ep*EVs and *E. tarda*. (**A**) PPI network of *Ep*EVs proteins and their interactions with *E. tarda* proteins was generated using the STRING database. (**B**) Key pathway enrichments of interacting proteins include (b1) protein export, (b2) microbial metabolism in diverse environments, and (b3) metabolic pathways of *Ep*EVs in association with *E. tarda*. Proteins identified from *Ep*EVs (1487) by HPLC-MS/MS were used to visualize the network of proteins interacting with *E. tarda* proteins. A total of 673 protein interactions were observed from the 1487 *Ep*EVs proteins.

PPIs of *Ep*EVs were determined using the STRING database by subjecting 1487 of identified *Ep*EVs proteins. Protein networking was mapped using the default settings of STRING where the host organism was selected as *E. tarda* (Fig. 4A). Out of the identified proteins, 673 interactions were predicted with *E. tarda* proteins showing their association and involvement with various biological and functional processes. Fig. 4B shows three of the identified key protein interactions important for protein export, microbial metabolism in various environments, and metabolic pathways, further suggesting *Ep*EVs potential for cargo transportation and metabolism. KEGG pathway enrichment analysis revealed that 12 pathways were enriched (Table S3), out of which both aminoacyl-tRNA biosynthesis and RNA degradation pathways had 100% of the observed gene count (16/16 and 8/8, respectively) in relation to the background genes. Furthermore, from the 12 enriched pathways five were related to metabolic processes which show insights into the potential metabolic activity of *Ep*EVs proteins.

### *In Vitro* and *In Vivo* Toxicity Analysis and Cellular Internalization of *Ep*EVs

**Fig. 5:**
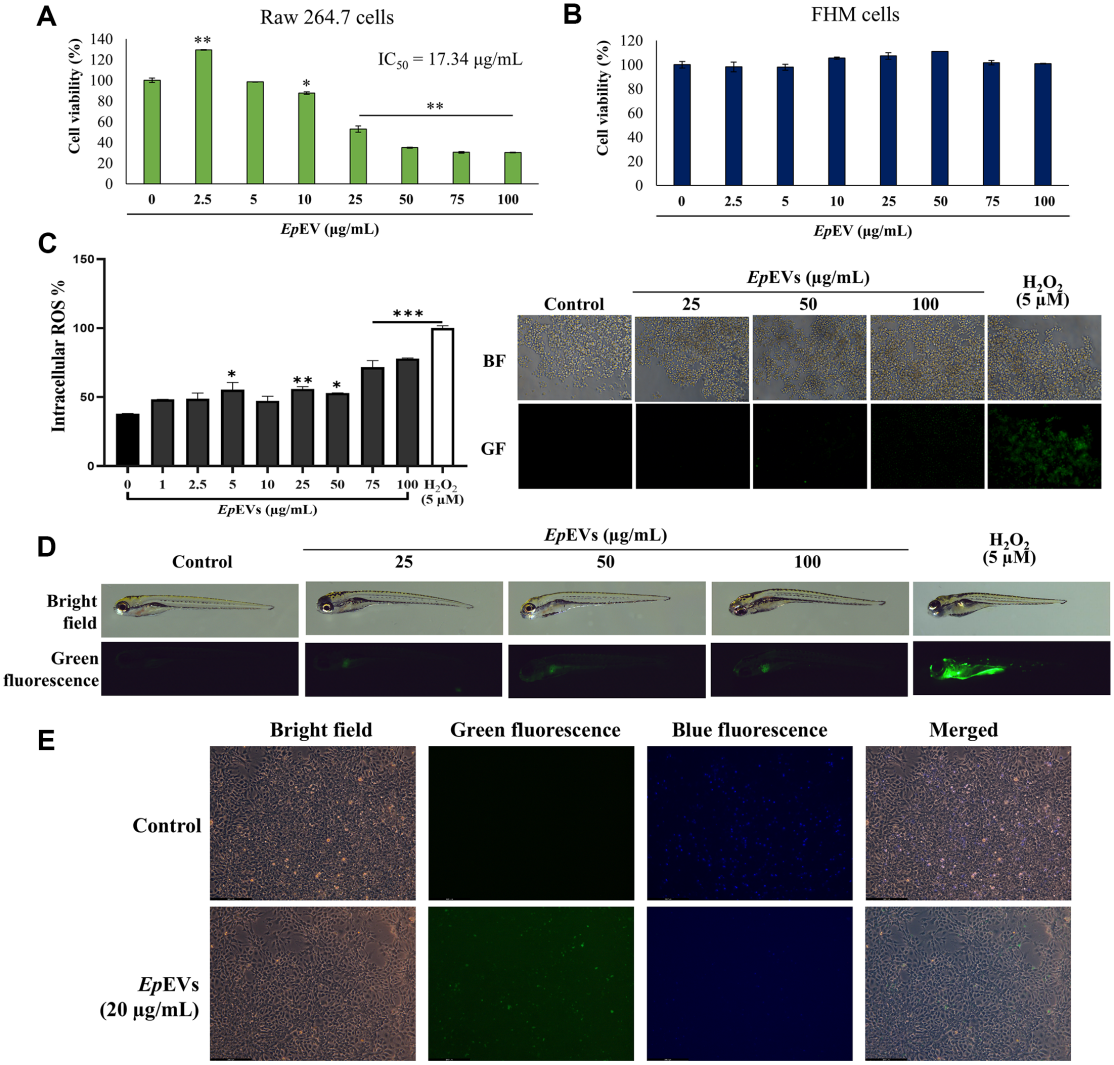
*In vitro* and *in vivo* toxic effects and internalization of *Ep*EVs. (**A**) Effect of *Ep*EVs on the viability of Raw 264.7 cells and (**B**) *Ep*EVs-treated FHM cells. Cytotoxicity in Raw 264.7 cells and FHM cells was assessed using the Cellrix cytotoxicity assay kit. (**C**) Intracellular ROS production was measured following treatment with DCFHDA (5 μg/ml), and representative images show the level of ROS production. (**D**) *In vivo* ROS generation in *Ep*EVs-treated zebrafish larvae (25-100 μg/ml). Zebrafish larvae at 60 hpf were treated with varying concentrations of *Ep*EVs, and mortality was measured up to 96 hpt. ROS generation was measured using DCFHDA stain, with H_2_O_2_ as the positive control. (**E**) Internalization of fluorescently labeled *Ep*EVs into FHM cells. Cells were analyzed by treating FHM cells (*n* = 3) with *Ep*EVs (20 μg/ml) using the ExoSparkler kit. Images were captured 24 hpt using a fluorescent microscope. Triplicate experiments were conducted to assess the repeatability of assays, and the data are presented as mean ± SEM.

The *in vitro* toxicity of *Ep*EVs was assessed using FHM and Raw 264.7 cells to understand the cell-specific toxicity of *Ep*EVs and to determine the safe dose for further experiments. At low concentrations of *Ep*EVs (below 10 μg/ml), Raw 264.7 cells did not show adverse effects (Fig. 5A). However, cells treated at 25 μg/ml showed a reduction of cell viability up to 52.85% with clear morphological changes (data not shown). Moreover, the viability of *Ep*EVs-treated Raw 264.7 cells was reduced drastically above 25 μg/ml. The IC_50_ value of *Ep*EVs was 17.34 μg/ml for Raw 264.7 cells. Next, we compared the toxicity of *Ep*EVs for FHM cells. Interestingly, *Ep*EVs did not demonstrate any apparent toxic effect even at 100 μg/ml (Fig. 5B) and we confirm IC_50_ value of *Ep*EVs for FHM cells could be above 100 μg/ml. Furthermore, intracellular ROS generation was analyzed in Raw 264.7 cells (Fig. 5C). Cellular ROS level was significantly increased (*p*<0.05) after 5 μg/ml *Ep*EVs treatment (no statistical significance for 10 μg/ml of *Ep*EVs) up to 100 μg/ml showing green fluorescent in cells compared to the negative control. Interestingly, *Ep*EVs treated cells exhibited less ROS generation compared to the positive control (5 μM; H_2_O_2_). Based on overall toxicity results safety dose for *in vitro* studies was confirmed as 5 and 10 μg/ml of *Ep*EVs for Raw 264.7 cells.

For the determination of *in vivo* toxicity, we determined the survival % of *Ep*EVs-treated zebrafish larvae at 60 hpf. No mortality was observed in *Ep*EVs-treated larvae (100 μg/ml) at 96 hpt (Data not shown). Additionally, we investigated whether *Ep*EVs treatment could induce oxidative stress in zebrafish larvae by analyzing *in vivo* ROS level (Fig. 5D). Comparatively low level of green fluorescence was detected only above 50 μg/ml treatment of *Ep*EVs and it was also lower than that of H_2_O_2_ (positive control) treated larvae group.

We used fluorescent-labeled *Ep*EVs to confirm cellular internalization into FHM cells (Fig. 5E). A preliminary experiment was conducted to optimize the peak time of internalization, and cells showed the highest fluorescence intensity at 24 hpt. The blue fluorescence in FHM cells is related to the DAPI-stained nuclei and the green fluorescence in FHM cells corresponds to internalized *Ep*EVs. Compared to the control, florescent labeled *Ep*EVs-treated FHM cells showed slight green fluorescence suggesting the successful internalization of *Ep*EVs at 24 hpt.

### *In Vitro* Immunomodulatory Activity of *Ep*EVs

**Fig. 6:**
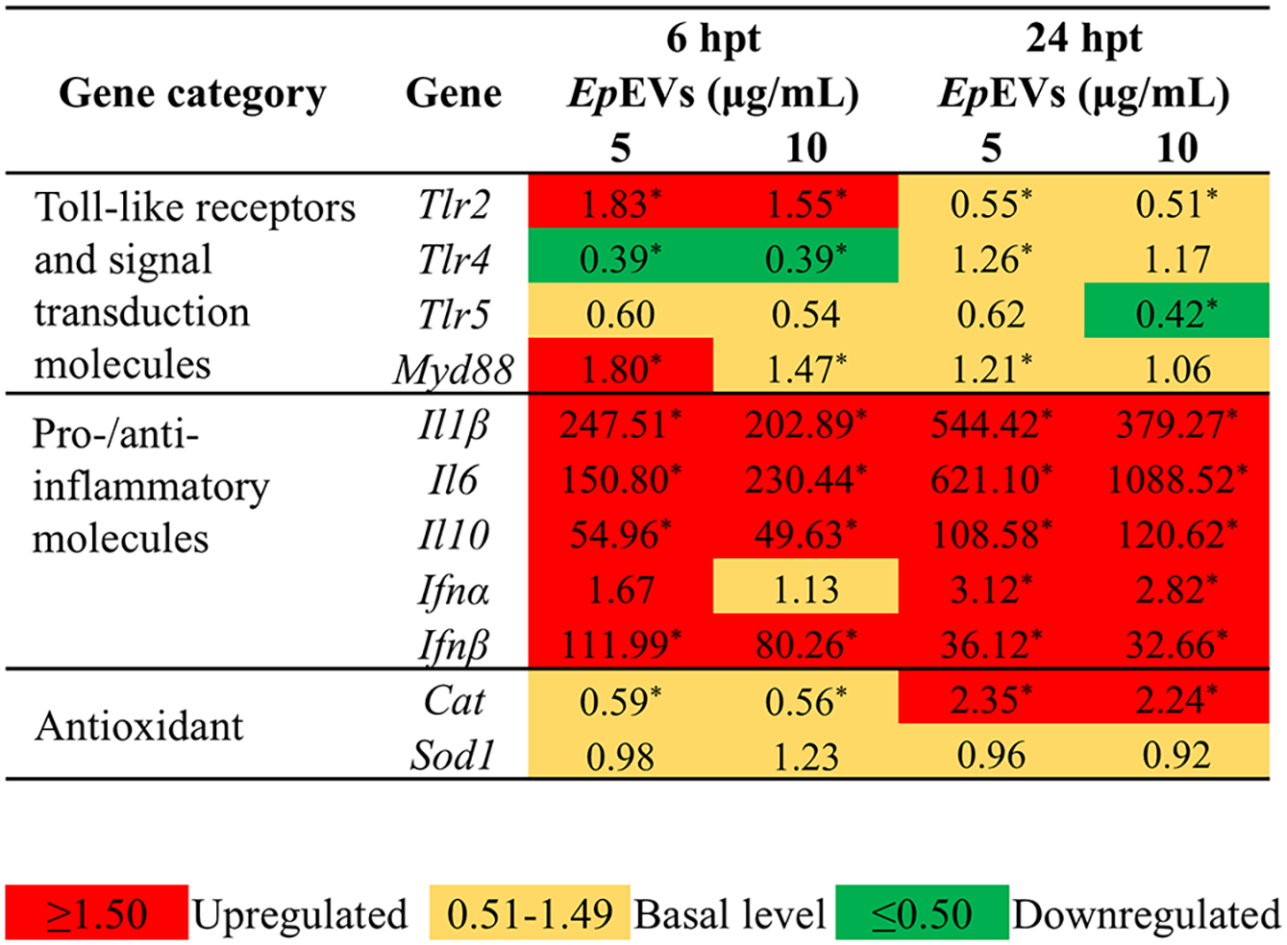
Analysis of transcriptional regulation in *Ep*EVs-treated Raw 264.7 cells. The transcriptional responses of Raw 264.7 cells to *Ep*EVs treatment were assessed using quantitative real-time PCR (qRT-PCR) to evaluate changes in gene expression. The expression levels of target genes were normalized to the housekeeping gene, *Gapdh*. The 2^−ΔΔCT^ method was applied to determine relative gene expression fold changes between *Ep*EVs-treated cells and untreated control cells. Data are presented as the mean relative expression fold change, calculated by comparing the expression levels in *Ep*EVs-treated cells to the control group. Statistical significance was determined at *p* < 0.05, with significant differences indicated by an asterisk (*)

To study whether *Ep*EVs have immunomodulatory effects, we used *Ep*EVs-treated Raw 264.7 cells and investigated the transcriptional regulation by qRT-PCR at 6 and 24 hpt (Fig. 6). Among the selected Toll-like receptors (Tlrs), only *Tlr2* was significantly upregulated (*p* < 0.05) upon both *Ep*EVs treatments (5 μg/ml; 1.83*-fold, and 10 μg/ml; 1.55*-fold) at 6 hpt. Myeloid differentiation primary response 88 (*Myd88*) was upregulated only at 5 μg/ml treatment of *Ep*EVs (6 hpt). Expression of other Tlrs such as *Tlr4* and *Tlr5* expressed almost basal levels except for *Tlr4* (6 hpt) and *Tlr5* (24 hpt) at 10 μg/ml. Pro and anti-inflammatory molecules including *Il1β*, *Il6*, and *Il10* were significantly upregulated (*p* < 0.05) in both concentrations and time-dependent manner. Moreover, *Il6* had the highest expression level (1088.52-fold at 10 μg/ml; 24 hpt). *Il1β* had the second highest relative fold expression at 24 hpt (5 μg/ml; 544.42*-fold and 10 μg/ml; 379.27*-folds). *Ifnα* was upregulated for both concentrations (5 μg/ml; 3.12-fold and 10 μg/ml; 2.82-fold) only at 24 hpt. *Ifnβ* expression levels were over 30-fold upregulated in both *Ep*EVs concentration and time points. Genes related to antioxidant enzymes including catalase (*Cat*) and superoxide dismutase1 (*Sod1*) were evaluated and results confirm the upregulated Cat expression at 24 hpt in both concentrations (5 μg/ml; 2.35*-fold, and 10 μg/ml; 2.24*-fold) while *Sod1* remained at a basal level for all time points. In comparison, *Il6* was the only gene that showed a concentration-dependent transcriptional upregulation at both time points.

### *In Vivo* Immunomodulatory Activity of *Ep*EVs

**Fig. 7:**
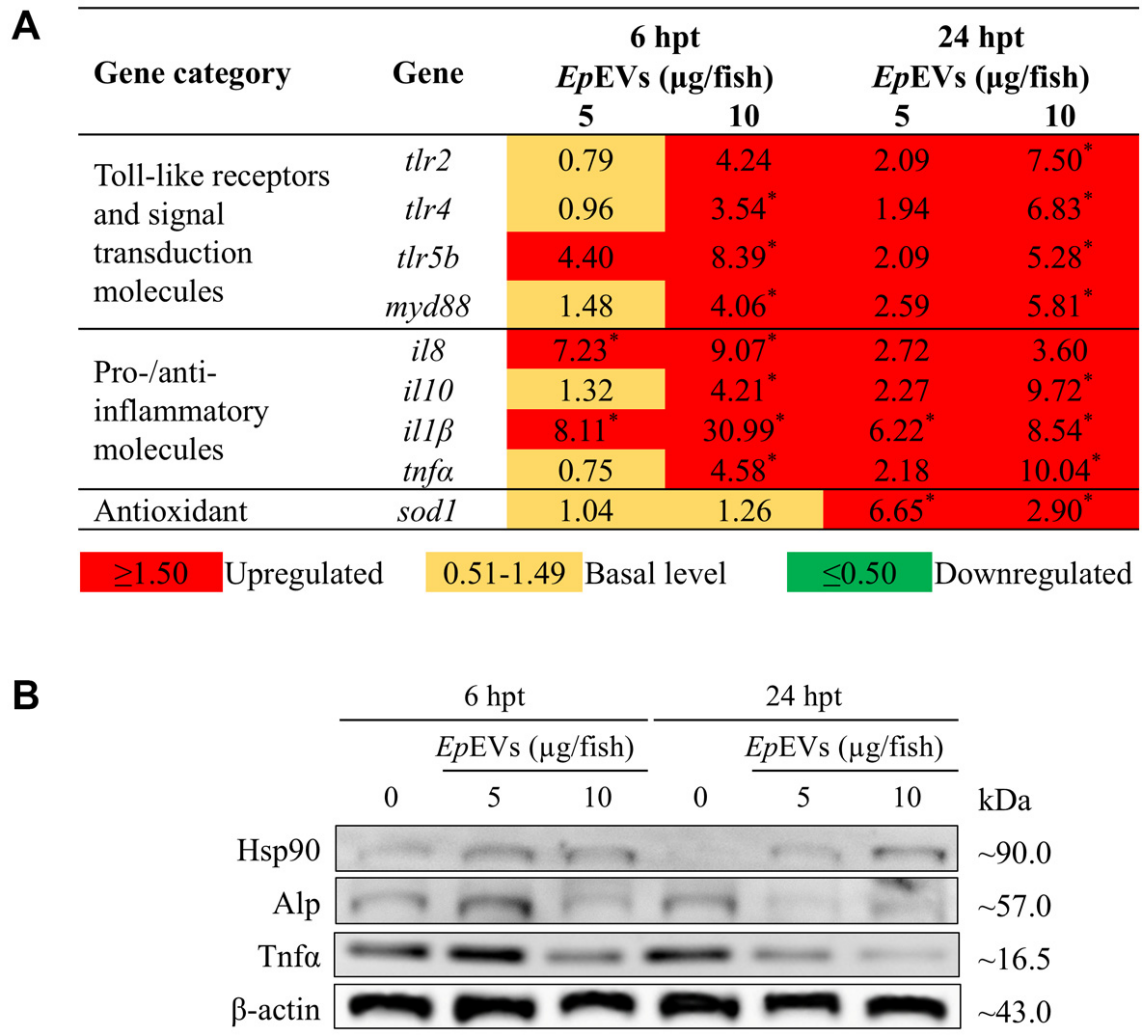
*In vivo* transcriptional expression and protein expression analysis of *Ep*EVs-treated zebrafish. (**A**) Transcriptional expression of immune-related functional genes in kidney. To assess the transcriptional expression of target genes, adult zebrafish were intraperitoneally injected with varying doses of *Ep*EVs (5 and 10 μg/fish). The relative fold expression of selected genes was quantified using the 2^−ΔΔCT^ method, with normalization to *β-actin* as the housekeeping gene. Statistical significance (*) was assessed at *p* < 0.05. (**B**) Analysis of protein expression in spleen tissue. Protein levels were assessed by western blotting. β-actin was used as the housekeeping protein to normalize protein loading across samples.

*In vivo*, immunomodulatory responses of *Ep*EVs were evaluated by isolating mRNA from *Ep*EVs-treated adult zebrafish kidney tissue by qRT-PCR (Fig. 7A). Overall, all the selected genes were upregulated in zebrafish with 5μg/fish of *Ep*EVs at both 6 and 24 hpt. However, at 6 hpt, 5 μg/fish *Ep*EVs treatment did not upregulate most genes (*tlr2*, *tlr4*, *myd88*, *il10*, and *tnfα*) which were upregulated in the 10 μg/fish *Ep*EVs-treated zebrafish. Additionally, *tlr5b* showed upregulation in both *Ep*EVs-treated groups. The *myd88* was upregulated only in the 10 μg/fish *Ep*EVs treatment group. At 24 hpt, expression of all *tlr* genes and *myd88* was increased. The highest fold change in *tlr* expression was observed for *tlr4* in the 10 μg/fish *Ep*EVs-treated group at 24 hpt. Both *il1β* and *il8* showed upregulation in response to *Ep*EVs treatment at both 6 and 24 hpt. Specifically, *il1β* exhibited the highest relative fold expression (30.99-fold) at 6 hpt in the 10 μg/fish treatment group. Antioxidant gene *sod1* remained at basal level at 6 hpt but showed upregulation at 24 hpt.

In addition, *in vivo* immunomodulation was evaluated using Western blotting analysis with spleen samples from adult zebrafish (Fig. 7B). Hsp90 levels were similarly expressed in both *Ep*EVs-treated groups at both time points. Alp levels were induced at 6 hpt with a reduction observed at 24 hpt. *Tnfα* expression decreased in the 10 μg/fish *Ep*EVs-treated group at 6 hpt and concentration-dependent reduction was observed at 24 hpt.

## Discussion

BEVs released by pathogenic bacteria could potentially be developed as vaccine candidates due to their ability to present bacterial-specific virulence factors, which can trigger immune responses in the host [10]. In this study, we applied the ultracentrifugation technique to isolate BEVs from *E. piscicida* as it is one of the least disruptive methods for isolating BEVs, according to Libardo *et al*. [24]. We confirmed the host bacterium *E. piscicida* shares 100% similarity with the known *E. piscicida* strain (accession no. JRGQ0100013) isolated by Camus *et al*.[25]. Next, we confirmed that *Ep*EVs are nanosized (85.3 ± 1.8 nm), spherical-shaped particles with clear margins, within the range described for BEVs (20-400 nm) [2]. The findings of Brudal *et al*. demonstrated that BEVs isolated from fish pathogenic bacterium *Francisella noatunensis* had an average size of 72.34 nm, which is relatively similar to our results [26]. However, Park *et al*. reported smaller-sized *E. tarda* EVs with an average diameter ranging from 10-40 nm [10]. The size difference may be attributed to the ultracentrifugation-based sucrose density gradient method used in the study. The major advantage of ultracentrifugation is its ability to yield a high quantity of EV extracellular vesicle particles with considerable purity. The negative charge of EVs is an overlooked characteristic that plays a vital role in their attachment to host cells and the internalization of cargo into EVs. According to Midekessa *et al*., the behavior of nanoscale molecules such as EVs, is highly influenced by their surface charge. Furthermore, the surface charge of EVs depends on factors such as the ionization level of membrane surface groups (*e.g.*, COO^-^ group of certain proteins), protonation, the presence of polymeric chains attached to the surface and H-bonds, types of intramolecular bonds, and the availability of ions in the solution [27]. Kaddour *et al*. reported that human blood-derived EVs (zeta-potential; −1.4 mV) were less efficiently internalized into human cervical epithelial cells, primary blood-derived monocytes, and primary peripheral blood lymphocytes compared to semen EVs (zeta-potential; −8.82 mV) [28]. Moreover, Kaddour *et al*. emphasized that the surface charge of EVs plays a crucial role in determining their attachment and internalization to target cells. Typically, gram negative BEVs exhibit a more pronounced negative charge compared to gram positive BEVs, primarily due to the presence of negatively charged phospholipids in the bacterial membrane of the respective strains [29]. However, the presence of cations such as Mg^2+^ and Ca^2+^ can stabilize the net charge, allowing BEVs to be stored long-term at -80°C without any damage [30]. Therefore, we proposed that *Ep*EVs could serve as a promising biomaterial to use as a vaccine candidate or drug delivery agent in fish medicine. NTA further confirms the efficiency of ultracentrifugation, showing a relatively higher particle concentration of 2.36 ± 0.11 × 10^12^ particles/ml and a protein recovery of 2.0 mg/l from the bacterial supernatant. Won *et al*. reported that culturing a large batch (200 L) of *Escherichia coli* yielded 357 mg (1.79 mg/l) of total protein amount of BEVs, equivalent to 3.93 × 10^15^ particles (1.96 × 10^10^ particles/mL of starting culture) [31]. In contrast, its final concentration was lower compared to our results. Therefore, it is important to explore methods for isolating BEVs at larger scales to enhance isolation efficiency while reducing costs.

Proteomic characterization of *Ep*EVs was conducted to gain deeper insights into their structure and function, as well as to understand how each component may contribute to the overall functionality of *Ep*EVs as a potential immunomodulatory agent or a vaccine candidate. Park *et al*. identified 74 proteins from outer membrane vesicles released by *E. tarda*, whereas the present study identified 1,487 proteins [10]. Out of the total *E. piscicida* proteome, this represents approximately 21.37%, indicating a significantly higher protein count and suggesting the presence of various heterogeneous proteins with diverse functions. Although it is the only available literature with similarities to *Ep*EVs, the isolation procedure and proteomic analysis methods used were different from those in the current study, which may explain the differing results. *Ep*EVs contain proteins such as hemolysin, Omps, and flagellin, Type III secretory system (T3SS) proteins, and chaperones all of which are essential for the pathogenesis of *E. piscicida* [32, 33]. Moreover, among the mentioned virulence factors, flagellin, and T3SS translocon protein were two of the five most abundant proteins identified in the total protein sample (data not shown). Results of the present study identified three T3SS proteins, two flagellin derivatives, nine Omps, their assembly factors, and 15 chaperone candidates. These components could be targeted in future studies to develop *Ep*EVs as a vaccine for controlling Edwardsiellosis. PPIs were predicted using the STRING database to construct a network of interactions between *Ep*EVs proteins and those of *E. tarda*. Furthermore, KEGG analysis revealed 12 enriched pathways in the predicted PPI network, with pathways related to various metabolic activities being highly enriched. Pathways such as aminoacyl-tRNA biosynthesis, protein export, and ribosome are known for their roles in protein synthesis and transportation [34]. Additionally, pathways related to purine metabolism, carbon metabolism, oxidative phosphorylation, metabolic pathways, biosynthesis of secondary metabolites, and microbial metabolism in diverse environments were identified as key pathways linked to various metabolic activities in the predicted PPIs. Together we provide important insights into how *Ep*EVs inherit and carry a substantial portion of the proteins from their originating organism, as well as how these proteins interact in relation to protein biosynthesis, metabolism, and transportation which are key processes to consider when evaluating *Ep*EVs as a vaccine candidate. However, it is important to note that further studies should focus on host PPIs with *Ep*EVs and their underlying mechanisms.

To further confirm the safety of *Ep*EVs as therapeutic agents, both *in vitro* and *in vivo* studies were conducted. The toxicity of *Ep*EVs was evaluated by treating Raw 264.7 murine macrophage cells, and FHM cells *in vitro* as well as zebrafish larvae *in vivo*. Results revealed IC_50_ of 17.34 μg/ml for *Ep*EVs in Raw 264.7 cells, while FHM cells showed no cytotoxicity even at a concentration of 100 μg/ml. In contrast, Vanaja *et al*. explained that the primary cause of toxicity in BEVs is the presence of LPS [35]. They further reported that *E. coli*-derived EVs (125 μg/ml) caused more than 50% cell death in bone marrow-derived mast cells, accompanied by an increase in cytosolic LPS levels. This phenomenon could explain the slight increase in toxicity observed when *Ep*EVs were treated to Raw 264.7 cells at higher concentrations. Furthermore, Matsuda *et al*. observed a 60% survival rate in zebrafish embryos at 96 hpt when treated with milk-derived EVs (2 × 10^12^ particles/ml) which is a relatively higher concentration than that used in the present study [36].

Excessive production of ROS can be detrimental to cells and tissues causing oxidative stress that damages the structural integrity and functionality of living organisms [37]. Therefore, measuring ROS generation provides a direct indication of the toxicity level of a sample when applied to cells and animals. According to the findings of Marzoog *et al*., *E. coli*-derived BEVs induced higher levels of ROS generation in a concentration-dependent manner (12.5-50 μg/ml) when treated with HT-29 human colon cancer cells, demonstrating their anti-cancer effect [38]. Overall, both *in vitro* and *in vivo* ROS generation showed no significant increase following *Ep*EVs treatment, further supporting the conclusion that isolated EVs exhibit lower toxicity. Therefore, the safety dose of *Ep*EVs was determined as ≤ 10 μg/ml for Raw 264.7 cells and ≤ 100 μg/ml for zebrafish.

As EVs become increasingly important in drug delivery for specialized therapies, cellular internalization of EVs plays a crucial role in transporting the specified cargo to the target organ [5]. Cellular internalization of BEVs occurs through one of five identified mechanisms; endocytosis, membrane fusion, receptor-mediated signaling, phagocytosis, and micropinocytosis [39]. Membrane fusion and endocytosis facilitate EVs to enter host cells by forming early endosomes, which release their cargo in a process that is heavily dependent on EV size [39, 40]. In receptor-mediated signaling, host cell receptors, such as pattern recognition receptors (Tlrs) are activated to mediate host cellular signaling for BEVs internalization, a process currently utilized in cancer therapy [39]. In this study, fluorescent labeling of *Ep*EVs was conducted by labeling the lipid bilayer, which is important for investigating their uptake by cells, biodistribution, and effect on cellular activities [41]. Additionally, it allows for the accurate characterization of individual EVs. The observed results confirm that *Ep*EVs can be fluorescently labeled and they are successfully internalized into FHM cells. This suggests their potential as a delivery system. Further modifications could optimize the loading of specific drugs into *Ep*EVs, as well as their release both *in vitro* and *in vivo*.

Although the fundamental basis of pathogenic bacteria is to infect the host without triggering its defense mechanisms, certain structural components of the bacteria can activate the host immune system to control the infection. Since BEVs have a membrane structure similar to that of parent bacteria, these molecules can stimulate the host immune system without causing infection [6]. Flagellin, LPS, and peptidoglycan are key bacterial membrane components commonly found in BEVs. These molecules act as virulence factors, mimicking the presence of bacteria and being recognized by pathogen recognition receptors (PRRs) [Tlrs, nucleotide-binding oligomerization domain-like receptors (NLR), C-type lectin receptors, and RIG-1 like receptors (RLR)] [42, 43]. This is consistent with our *in vitro* and *in vivo* transcriptional analysis results. While Raw 264.7 cells showed upregulation of only *Tlr2* (at 6 hpt), upregulation of *tlr2*, *tlr4*, and *tlr5b* in zebrafish was observed for 24 hpt for both concentrations suggesting that *Ep*EVs have the ability to stimulate host Tlrs.

Generally, the stimulation of host PRRs activates various transcription factors, which regulate cell signaling pathways, mediating the host’s innate immune responses and triggering the release of a range of pro-inflammatory (Tnf-α, *Il1β*, *Il6*, and Il8) and anti-inflammatory (*Il10*) cytokines [43]. These cytokines further activate immune cells, priming and maturing them to produce molecules that amplify the inflammatory responses, while also stimulating the production of type 1 Ifns, antimicrobial peptides, and chemokines that help control or mitigate the ongoing threat to the host and provide protection [43, 44]. The results of the present study show reveal that at 24 hpt both concentrations of *Ep*EVs (5 and 10 μg/ml) induced upregulation for pro and anti-inflammatory cytokines in zebrafish and Raw 264.7 cells consistent with previous reports. Sivanantham *et al*. have demonstrated that *E. coli*-derived EVs (0.25 μg/ml) have activated *Tlr4*, which led to the release of *Tnfα* and *Il1β* pro-inflammatory cytokines in murine bone marrow-derived macrophage cells [42]. Moreover, a study conducted by Lagos *et al*. shows that EVs from *F. noatunensis* subsp. *orientalis* increased the expression of pro-inflammatory cytokines *tnfα*, *il1β*, and *ifnγ* which aligns with our findings [45]. In addition, a study conducted by Park *et al*. demonstrated the elevation of Tlr2, Il1β, Il6, TNFα, and IFNγ upon treatment of *E. tarda* (at present *E. piscicida*)-derived EVs at 0.2 μg/g of body weight when injected to olive flounders [10]. When compared to the treatment doses (~10 and 20 μg/g of body weight of fish) of the present study, the expression level was relatively lower, which could be due to other factors such as the use of different fish species, environmental factors, stress conditions as well as the age. Alp is a group of enzymes that catalyze the hydrolytic removal of phosphate ions from various molecules [46]. Specifically, they play a role in the detoxification of LPS derived from the outer membrane of gram-negative bacteria, thereby modulating host-bacterial interactions [46]. Hsp90 is a universally expressed protein, except in archaea, and plays a crucial role in regulating cell survival [47]. A study conducted by Bates *et al*. revealed that intestinal Alp-deficient zebrafish were hypersensitive to LPS and exhibited elevated expression of *myd88* and *tnfγ*, suggesting an active inflammatory response [48]. Therefore, since Alp protein levels were comparatively high at 6 hpt, it can be assumed that Alps contributed to reducing the inflammatory response, while Hsp90 is important for cell survival and stress response. Furthermore, Cecil *et al*. reported that BEVs released from *Porphyromonas gingivalis* can trigger an initial pro-inflammatory response characterized by the production of TNFα and Il1β, which primes monocytes and macrophages. A subsequent exposure, however, induces the release of the anti-inflammatory *Il10*, promoting tolerance to endotoxins [49]. This finding may further support the results observed in the present study, which demonstrated the simultaneous expression of both pro-inflammatory (*Il1β*, *Il6*) and anti-inflammatory (*Il6*) cytokines. Cat and Sods are essential enzymes that activate inherent anti-oxidative mechanisms to minimize oxidative stress caused by stimulated inflammation [50]. Even though ROS production was relatively low when *Ep*EVs were treated with Raw 264.7 cells and zebrafish larvae, oxidative stress resulting from immune response at 24 hpt was evident, as indicated by the high expression of *Cat* in Raw 264.7 cells and *sod1* in zebrafish kidney tissues. Although cellular *Sod1* remained at the basal level, *Cat* expression was upregulated at 24 hpt, likely due to increased levels of peroxide ions in the form of H_2_O_2_. Cat primarily converts H_2_O_2_ into H_2_O while comparatively higher toxic superoxide free radicals are managed by Sods [51]. Based on this, we can hypothesize that treatment with *Ep*EVs may increase the H_2_O_2_ in Raw 264.7 cells, leading to an oxidative stress response. Al-Nedawi *et al*. reported that *Lactobacillus rhamnosus*-derived EVs induced *Il10* and heme-oxygenase-1 expression in dendritic cells [52]. This finding may be linked to our *in vitro* and *in vivo* observations, where *Il10* upregulation, in combination with increased levels of *Cat* and *Sod1*, regulated both the inflammatory response and oxidative stress simultaneously.

## Conclusion

This study demonstrates that ultracentrifugation is an effective and convenient technique for isolating high-purity BEVs from gram negative *E. piscicida*, preserving their characteristic properties. Through comprehensive proteomic analysis, we identified specific proteins consistently present in BEVs, providing valuable insight into their biological functions. Additionally, we identified several common proteins as cargo molecules in *Ep*EVs which may play crucial roles in intercellular communications and the transfer of biological information. Our findings demonstrate that *Ep*EVs have been uptaken by cells. However, further research is needed to investigate mechanisms underlying the internalization of *Ep*EVs and the functional outcomes of their uptaken in various cellular contexts. Furthermore, *Ep*EVs exhibited minimal toxicity when tested with Raw 264.7 cells and zebrafish larvae. The immunomodulatory effects of *Ep*EVs were highly promising when treated to Raw 264.7 cells and zebrafish. Overall, these results have significant implications for the development of *Ep*EVs-based therapeutic strategies, including vaccines and drug delivery systems in fish medicine.

## Supplemental Materials

Supplementary data for this paper are available on-line only at http://jmb.or.kr.



## Figures and Tables

**Table 1:** Proteomic studies on BEVs derived from fish pathogenic bacteria: Selected highlights.

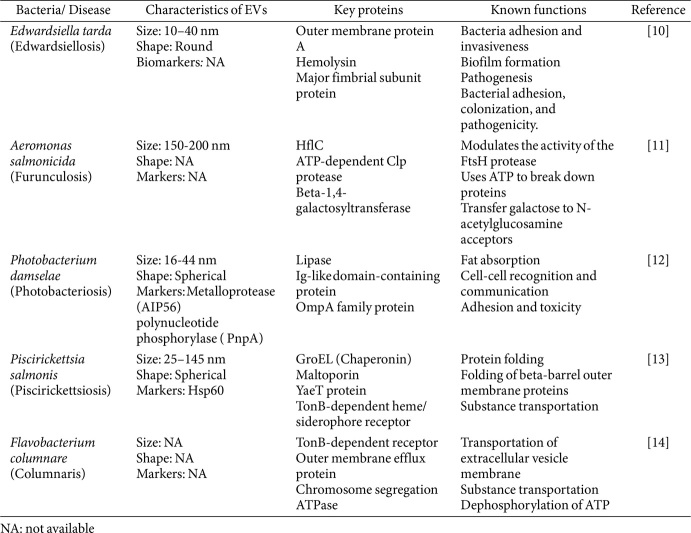

**Table 2:** Description of antibodies used in this study.

Primary antibody	Manufacturer/Reference No.	Dilution	Secondary antibody
Flagellin	Abcam, Gyeonggi do, Republic of Korea (ab93713)	1:10000	Anti-rabbit IgG - HRP
Outer membrane protein A (OmpA)	Antibody Research Corporation, St. Peters, MO, USA (111120)	1:50000	Anti-rabbit IgG - HRP
Heat shock protein 90 (Hsp90)	Cell signaling Technology, MA, USA (4874)	1:1000	Anti-rabbit IgG - HRP
Alkaline phosphatase (Alp)	GeneTex, Quebec, Canada (GTX112100)	1:1000	Anti-rabbit IgG - HRP
Tumor necrosis factor α (Tnfα)	Kingfisher Biotech, Inc, MI, USA (KP1540Z-100)	1:1000	Anti-rabbit IgG - HRP
Βeta-actin (β actin)	Santa Cruz Biology Inc, OR, USA (Sc-4778)	1:1000	Anti-mouse IgG - HRP

**Table 3:** Description of primers used in this study.

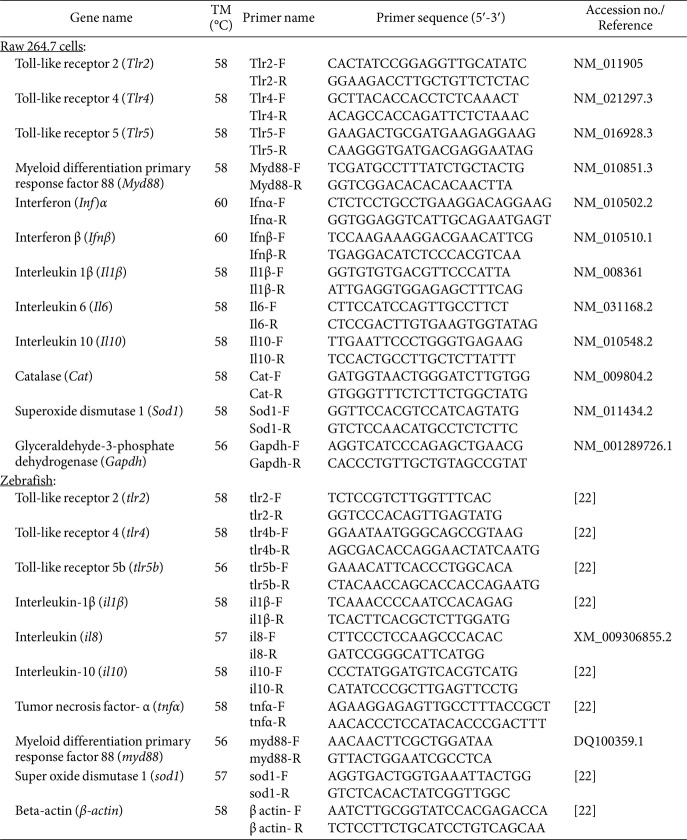

**Table 4:** Summary of key proteins associated with *Ep*EVs and their functions.

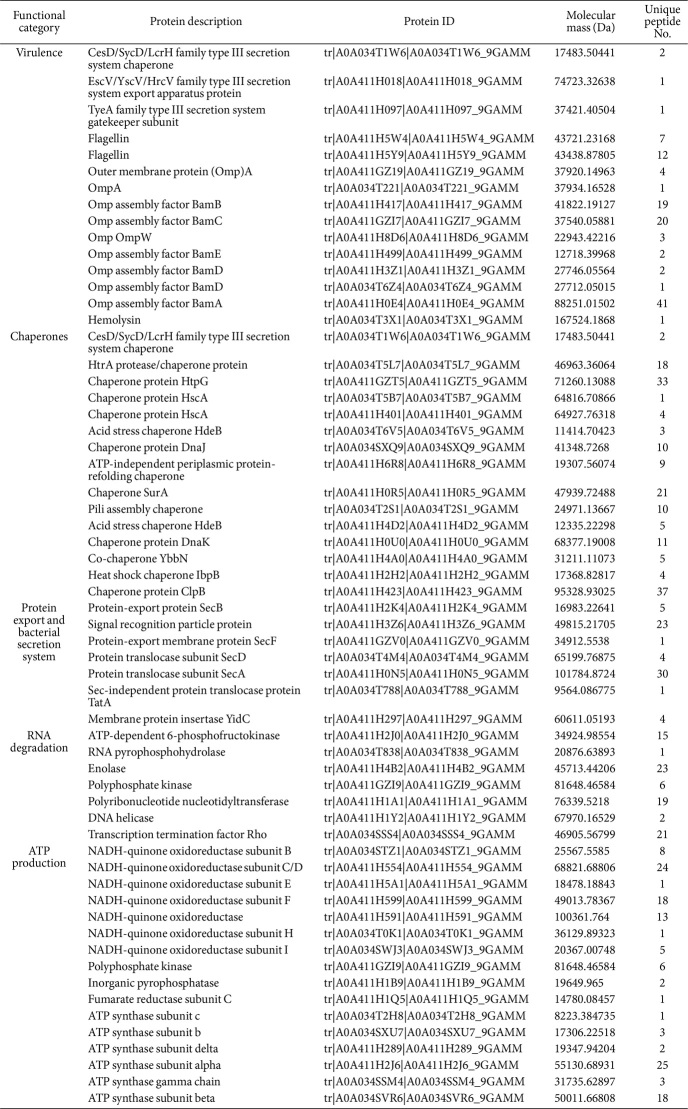

## References

[1] Du S, Guan Y, Xie A, Yan Z, Gao S, Li W (2023). Extracellular vesicles: a rising star for therapeutics and drug delivery. J. Nanobiotechnol..

[2] Liu H, Zhang Q, Wang S, Weng W, Jing Y, Su J (2022). Bacterial extracellular vesicles as bioactive nanocarriers for drug delivery: advances and perspectives. Bioact. Mater..

[3] Wen M, Wang J, Ou Z, Nie G, Chen Y, Li M (2023). Bacterial extracellular vesicles: a position paper by the microbial vesicles task force of the Chinese society for extracellular vesicles. Interdisciplinary Medicine..

[4] Xie J, Haesebrouck F, Van Hoecke L, Vandenbroucke RE (2023). Bacterial extracellular vesicles: an emerging avenue to tackle diseases. Trends Microbiol..

[5] Matinha-Cardoso J, Coutinho F, Lima S, Eufrásio A, Carvalho AP, Oliva-Teles A (2022). Novel protein carrier system based on cyanobacterial nano-sized extracellular vesicles for application in fish. Microb. Biotechnol..

[6] Hosseini-Giv N, Basas A, Hicks C, El-Omar E, El-Assaad F, Hosseini-Beheshti E (2022). Bacterial extracellular vesicles and their novel therapeutic applications in health and cancer. Front. Cell. Infect. Microbiol..

[7] Chen C, Kawamoto J, Kawai S, Tame A, Kato C, Imai T (2020). Isolation of a novel bacterial strain capable of producing abundant extracellular membrane vesicles carrying a single major cargo protein and analysis of its transport mechanism. Front. Microbiol..

[8] Cho WC (2007). Proteomics technologies and challenges. Genomics Proteomics Bioinformatics.

[9] Zubair M, Wang J, Yu Y, Faisal M, Qi M, Shah AU (2022). Proteomics approaches: a review regarding an importance of proteome analyses in understanding the pathogens and diseases. Front. Vet. Sci..

[10] Park SB, Jang HB, Nho SW, Cha IS, Hikima J-i, Ohtani M (2011). Outer membrane vesicles as a candidate vaccine against edwardsiellosis. PLoS One.

[11] Kroniger T, Mehanny M, Schlüter R, Trautwein-Schult A, Köllner B, Becher D (2022). Effect of iron limitation, elevated temperature, and florfenicol on the proteome and vesiculation of the fish pathogen *Aeromonas salmonicida*. Microorganisms.

[12] Teixeira A, Loureiro I, Lisboa J, Oliveira PN, Azevedo JE, dos Santos NMS (2023). Characterization and vaccine potential of outer membrane vesicles from *Photobacterium damselae* subsp. *Piscicida*. Int. J. Mol. Sci..

[13] Oliver C, Valenzuela K, Hernández M, Sandoval R, Haro RE, Avendaño-Herrera R (2016). Characterization and pathogenic role of outer membrane vesicles produced by the fish pathogen *Piscirickettsia salmonis* under *in vitro* conditions. Vet. Microbiol..

[14] Liu GY, Nie P, Zhang J, Li N (2008). Proteomic analysis of the sarcosine-insoluble outer membrane fraction of *Flavobacterium columnare*. J. Fish Dis..

[15] Leung KY, Wang Q, Yang Z, Siame BA (2019). *Edwardsiella piscicida*: a versatile emerging pathogen of fish. Virulence.

[16] Jung WJ, Kwon J, Giri SS, Kim SG, Kim SW, Kang JW (2022). Isolation and characterization of a highly virulent *Edwardsiella piscicida* strain responsible for mass mortality in marbled eel (*Anguilla marmorata*) cultured in Korea. Aquaculture.

[17] Mohanty BR, Sahoo PK (2007). Edwardsiellosis in fish: a brief review. J. Biosci..

[18] Jayathilaka EHTT, Rajapaksha DC, Nikapitiya C, De Zoysa M, Whang I (2021). Antimicrobial and anti-biofilm peptide octominin for controlling multidrug-resistant *Acinetobacter baumannii*. Int. J. Mol. Sci..

[19] Nikapitiya C, Jayathilaka EHTT, Edirisinghe SL, Rajapaksha DC, Wasana WP, Jayasinghe JNC (2022). Isolation and characterization of plasma-derived exosomes from the marine fish rock bream (*Oplegnathus fasciatus*) by two isolation techniques. Fishes.

[20] Jayathilaka EHTT, Edirisinghe SL, Lee J, Nikapitiya C, De Zoysa M (2022). Isolation and characterization of plasma-derived exosomes from olive flounder (*Paralichthys olivaceus*) and their wound healing and regeneration activities. Fish Shellfish Immunol..

[21] Jayathilaka EHTT, Dias MKHM, Tennakoon MSBWTMNS, Chulhong O, Nikapitiya C, Shin H-J (2024). Mapping the proteomic landscape and anti-inflammatory role of *Streptococcus parauberis* extracellular vesicles. Fish Shellfish Immunol..

[22] Edirisinghe SL, Dananjaya SHS, Nikapitiya C, Liyanage TD, Lee K-A, Oh C (2019). Novel pectin isolated from *Spirulina maxima* enhances the disease resistance and immune responses in zebrafish against *Edwardsiella piscicida* and *Aeromonas hydrophila*. Fish Shellfish Immunol..

[23] Livak KJ, Schmittgen TD (2001). Analysis of relative gene expression data using real-time quantitative PCR and the 2^−ΔΔCT^ method. Methods.

[24] Dias MKHM, Jayathilaka EHTT, De Zoysa M (2024). Isolation, characterization, and immunomodulatory effects of extracellular vesicles isolated from fish pathogenic Aeromonas hydrophila. Fish Shellfish Immunol..

[25] Libardo MDJ, Durr E, Hernandez LD (2023). A robust protocol to isolate outer membrane vesicles from nontypeable *Haemophilus influenzae*. Methods Protoc..

[26] Camus A, Griffin M, Armwood A, Soto E (2019). A spontaneous outbreak of systemic *Edwardsiella piscicida* infection in largemouth bass *Micropterus salmoides* (Lacépède, 1802) in California, USA. J. Fish Dis..

[27] Brudal E, Lampe EO, Reubsaet L, Roos N, Hegna IK, Thrane IM (2015). Vaccination with outer membrane vesicles from *Francisella noatunensis* reduces development of francisellosis in a zebrafish model. Fish Shellfish Immunol..

[28] Midekessa G, Godakumara K, Ord J, Viil J, Lättekivi F, Dissanayake K (2020). Zeta potential of extracellular vesicles: toward understanding the attributes that determine colloidal stability. ACS Omega.

[29] Kaddour H, Panzner TD, Welch JL, Shouman N, Mohan M, Stapleton JT (2020). Electrostatic surface properties of blood and semen extracellular vesicles: implications of sialylation and HIV-induced changes on EV internalization. Viruses.

[30] Wei S, Jiao D, Xing W (2022). A rapid method for isolation of bacterial extracellular vesicles from culture media using epsilon-poly-L-lysine that enables immunological function research. Front. Immunol..

[31] Ñahui Palomino RA, Vanpouille C, Costantini PE, Margolis L (2021). Microbiota-host communications: bacterial extracellular vesicles as a common language. PLOS Pathog..

[32] Won S, Lee C, Bae S, Lee J, Choi D, Kim MG (2023). Mass-produced gram-negative bacterial outer membrane vesicles activate cancer antigen-specific stem-like CD8(+) T cells which enables an effective combination immunotherapy with anti-PD-1. J. Extracell Vesicles.

[33] Liu YL, He TT, Liu LY, Yi J, Nie P, Yu HB (2019). The *Edwardsiella piscicida* Type III translocon protein EseC inhibits biofilm formation by sequestering EseE. Appl. Environ. Microbiol..

[34] Cao H, Yang C, Quan S, Hu T, Zhang L, Zhang Y (2018). Novel T3SS effector EseK in *Edwardsiella piscicida* is chaperoned by EscH and EscS to express virulence. Cell Microbiol..

[35] Rubio Gomez MA, Ibba M (2020). Aminoacyl-tRNA synthetases. RNA (New York, NY.).

[36] Vanaja SK, Russo AJ, Behl B, Banerjee I, Yankova M, Deshmukh SD (2016). Bacterial outer membrane vesicles mediate cytosolic localization of LPS and caspase-11 activation. Cell.

[37] Matsuda A, Moirangthem A, Angom RS, Ishiguro K, Driscoll J, Yan IK (2020). Safety of bovine milk derived extracellular vesicles used for delivery of RNA therapeutics in zebrafish and mice. J. Appl. Toxicol..

[38] Chatterjee S, Thomas Dziubla DAB (2016). Oxidative Stress and Biomaterials.

[39] Marzoog TR, Jabir MS, Ibraheem S, Jawad SF, Hamzah SS, Sulaiman GM (2023). Bacterial extracellular vesicles induced oxidative stress and mitophagy through mTOR pathways in colon cancer cells, HT-29: implications for bioactivity. Biochim. Biophys. Acta Mol. Cell Res..

[40] Liu H, Zhang H, Han Y, Hu Y, Geng Z, Su J (2022). Bacterial extracellular vesicles-based therapeutic strategies for bone and soft tissue tumors therapy. Theranostics.

[41] Gao L, van der Veen S (2020). Role of outer membrane vesicles in bacterial physiology and host cell interactions. MID.

[42] Dehghani M, Gulvin SM, Flax J, Gaborski TR (2019). Exosome labeling by lipophilic dye PKH26 results in significant increase in vesicle size. bioRxiv.

[43] Sivanantham A, Alktaish W, Murugeasan S, Gong B, Lee H, Jin Y (2023). Caveolin-1 regulates OMV-induced macrophage proinflammatory activation and multiple Toll-like receptors. Front. Immunol..

[44] Suri K, D'Souza A, Huang D, Bhavsar A, Amiji M (2023). Bacterial extracellular vesicle applications in cancer immunotherapy. Bioact. Mater..

[45] Mancini F, Rossi O, Necchi F, Micoli F (2020). OMV vaccines and the role of TLR agonists in immune response. Mol. Sci..

[46] Lagos L, Tandberg JI, Repnik U, Boysen P, Ropstad E, Varkey D (2017). Characterization and vaccine potential of membrane vesicles produced by *Francisella noatunensis* subsp. Orientalis in an adult zebrafish model. CVI..

[47] Yang Y, Wandler AM, Postlethwait JH, Guillemin K (2012). Dynamic evolution of the LPS-detoxifying enzyme intestinal alkaline phosphatase in zebrafish and other vertebrates. Front. Immunol..

[48] Hu C, Yang J, Qi Z, Wu H, Wang B, Zou F (2022). Heat shock proteins: biological functions, pathological roles, and therapeutic opportunities. MedComm..

[49] Bates JM, Akerlund J, Mittge E, Guillemin K (2007). Intestinal alkaline phosphatase detoxifies lipopolysaccharide and prevents inflammation in zebrafish in response to the gut microbiota. Cell Host Microbe.

[50] Cecil JD, O'Brien-Simpson NM, Lenzo JC, Holden JA, Singleton W, Perez-Gonzalez A (2017). Outer membrane vesicles prime and activate macrophage inflammasomes and cytokine secretion In vitro and *In vivo*. Front. Immunol..

[51] Younus H (2018). Therapeutic potentials of superoxide dismutase. Int. J. Health Sci..

[52] Weydert CJ, Cullen JJ (2010). Measurement of superoxide dismutase, catalase and glutathione peroxidase in cultured cells and tissue. Nat. Protoc..

[53] Al-Nedawi K, Mian MF, Hossain N, Karimi K, Mao YK, Forsythe P (2015). Gut commensal microvesicles reproduce parent bacterial signals to host immune and enteric nervous systems. FASEB J..

