# Supplementary material for: Exploring the Proteomic Landscape and Immunomodulatory Functions of Edwardsiella piscicida Derived Extracellular Vesicles
Source: J Microbiol Biotechnol. 2024 Dec 17;35:e2410001. doi: 10.4014/jmb.2410.10001 (PMC11813346; doi:10.4014/jmb.2410.10001)
Supplement: Supplementary file 1 [file jmb-35-e2410001-supple.pdf]

J. Microbiol. Biotechnol. 2025. 35: e2410001

https://doi.org/10.4014/jmb.2410.10001

Supplementary Tables

Table 1. **Flagellin and OmpA abundance in *Ep*EVs.**

Protein ID	Description	Gene Name	Protein Mass	iBAQ*
395_1	Flagellin	EVK84_14340	43721.23	8,369,450.0
395_2	Flagellin	EVK84_14345	43438.88	54,227,652.0
1198_1	Outer membrane protein A	ompA	37934.17	44,921.5
1198_2	Outer membrane protein A	ompA	37920.15	728,179.6

* iBAQ - Intensity-based absolute quantification (a measure of protein abundance)

Table S2. **Summary of proteomic analysis of *Ep*EVs.**

Characteristics	Number
Total spectra	56,179
Identified spectra	15,911
Identified peptides	10,745
Unique peptides	9,157
Identified proteins	1,487

Table S3. Summary of identified proteins in *Ep*EVs by KEGG pathway enrichment analysis.

#Term ID	Term description	Observed gene count	Background gene count	Strength	False discovery rate	Matching proteins in the network (labels)
etr00970	Aminoacyl-tRNA biosynthesis	16	16	0.660	6.60E-04	glyQ, trpS, valS, hisS, leuS, glnS, serS, pheS, thrS, aspS, argS, asnS, metG, gltX, ileS, proS
etr03018	RNA degradation	8	8	0.660	3.04E-02	pfkA, rppH_1, eno, ppk1, pnp, groEL, RecQ, rho
etr03010	Ribosome	42	44	0.640	1.22E-09	rpmE, rpsL, rpsG, rpsJ, rplC, rplD, rplW, rplB, rplV, rpsC, rplP, rpmC, rpsQ, rplN, rplX, rplE, rpsN, rpsH, rplF, rplR, rplO, rpsD, rplQ, rpsP, rplS, rplT, rpmI, rpsB, rpsI, rplM, rpsU, rpsO, rpmA, rplU, rplI, rpsR, rpsF, rplJ, rplA, rplK, rpmG, rpmB
etr03060	Protein export	9	11	0.570	3.73E-02	secB, ffh, secF, secD, yajC, secA, secE, tatA, yidC
etr03070	Bacterial secretion system	9	11	0.570	3.73E-02	secB, ffh, secF, secD, yajC, secA, secE, tatA, yidC
etr00030	Pentose phosphate pathway	10	14	0.520	3.75E-02	pfkA, tkt, rpiA, zwf, prs, tal, deoB, deoC, fbp, pgi
etr00230	Purine metabolism	16	24	0.490	9.40E-03	spoT, gmk, arcC, guaB, relA, pyk, prs, adk, apt, gpt, hpt, guaC, deoD, deoB, NrdD, purA
etr01200	Carbon metabolism	23	37	0.460	2.20E-03	pfkA, tpiA, arcC, accC, tkt, pgk, rpiA, glyA, eno, sucC, ackA, zwf, pyk, prs, accA, lpdA, aceE, tal, mdh, fbp, frdC, pgi, ppc
etr00190	Oxidative phosphorylation	16	27	0.430	2.05E-02	nuoB, nuoC, NuoE, NuoF, NuoG, nuoH, nuoI, ppk1, ppa, frdC, atpE, atpF, atpH, atpA, atpG, atpD
etr01110	Biosynthesis of secondary metabolites	44	81	0.400	5.23E-05	pfkA, tpiA, glgC, accC, glpC, metK, tkt, pgk, rpiA, glyA, ispG, guaB, hemL, eno, sucC, menB, fabG, acpP, ribA, AdhE, galU, zwf, pyk, prs, gltX, adk, ribH, proB, gpt, accA, cdsA, hpt, lpdA, aceE, tal, deoD, mdh, fbp, frdC, plsB, pgi, hemB, ubiE, ilvE
etr01100	Metabolic pathways	101	190	0.390	4.14E-11	spoT, gmk, pfkA, tpiA, glpK, glgC, arcC, accC, pepA, metK, speA, speB, tkt, pgk, rpiA, glyA, iscS, ispG, guaB, hemL, relA, pyrG, eno, lipA, DacA, glnS, sucC, ackA, nuoB, nuoC, NuoE, NuoF, NuoG, nuoH, nuoI, menB, fruK, pflB, fabH, fabG, acpP, ribA, dsdA, kduD, AdhE, galU, selD, FadD, zwf, pyk, kdsA, prs, pyrD, udk, metG, gltX, napA, upp, adk, apt, ribH, proB, gpt, accA, fabZ, cdsA, pyrH, hpt, lpdA, aceE, guaC, lpxC, murC, carB, tal, murA, deoD, deoB, deoC, NrdD, glmM, mdh, fbp, purA, frdC, aspA, torC, plsB, pgi, hemB, ubiE, ilvE, rfaD, sthA, ppc, atpE, atpF, atpH, atpA, atpG, atpD
etr01120	Microbial metabolism in diverse environments	30	62	0.350	5.50E-03	pfkA, tpiA, arcC, accC, tkt, pgk, rpiA, glyA, hemL, eno, sucC, ackA, pflB, AdhE, zwf, pyk, prs, gltX, napA, accA, lpdA, aceE, tal, mdh, fbp, frdC, torC, pgi, hemB, ppc
